# A massive right Sylvian fissure epidermoid cyst presented with progressive truncal ataxia and hemiparesis: a case report

**DOI:** 10.1097/MS9.0000000000005392

**Published:** 2026-08-06

**Authors:** Layal Abou Zeki, Zeinab Nahle, Marwan Haddad, Omar Al Awar

**Affiliations:** a Department of Neurosurgery, University of Balamand (UOB), Beirut, Lebanon; b Department of Neurosurgery, Lebanese American University (LAU), Beirut, Lebanon; c Department of Neurosurgery, Mount Lebanon Hospital, Beirut, Lebanon; d Mount Lebanon Hospital, Ain wa Zain medical Village and Al Jabal Hospital

**Keywords:** ataxia, case report, epidermoid cyst, surgical resection

## Abstract

**Introduction:**

Intracranial epidermoid cysts are rare congenital lesions that can occur in the suprasellar region, the cerebellopontine angle, the basilar-pontine fossa, the ventricular system, and, only rarely, the Sylvian fissure.

**Case presentation:**

We present the case of a 41 year-old male patient experiencing headache, nausea, vomiting, confusion, truncal ataxia, seizures, and left hemiparesis, who was found to have a right Sylvian fissure epidermoid cyst.

**Clinical discussion:**

A craniotomy was performed, and step-by-step intraoperative guidance was illustrated. Postoperative follow-up showed a normal neurological examination and resolution of the previously mentioned symptoms (seizures, headaches, nausea, vomiting, ataxia, confusion, left-sided weakness). A comparison with other cases of Sylvian fissure epidermoid cysts was conducted.

**Conclusion:**

An epidermoid cyst, although a benign entity, can present with serious symptoms when occurring in the Sylvian fissure, affecting patients’ quality of life and necessitating early diagnosis and intervention.

## Introduction

Intracranial epidermoid cysts are rare congenital lesions derived from the ectoderm, formed during embryogenesis, and represent 0.3–1.8% of all intracranial tumors^[^1,2^]^. They are slow-growing lesions and are mainly asymptomatic until they compress an eloquent brain structure^[^3^]^. Epidermoid cysts usually occur in the suprasellar, the cerebellopontine angle, the basilar-pontine fossa, within the ventricular system, and the Sylvian fissure^[^1^]^. Approximately, half of epidermoid cysts occur within the cerebellopontine angle, while in the Sylvian fissure they are extremely rare. After obtaining approval from our hospital and the patient, we report a case of a 40-year-old man with a large epidermoid cyst located in the right Sylvian fissure who presented with a seizure, headache, confusion (related to the postictal phase), truncal ataxia, and left hemiparesis. He underwent craniotomy and near-total resection of the tumor contents, with resolution of his symptoms. Such a location and presentation are rarely encountered for epidermoid tumors. Our literature review revealed only a few cases of middle fossa epidermoid tumors with a similar clinical presentation^[^4^]^. This case has been reported in line with the SCARE checklist^[^5^]^.

HIGHLIGHTS

- An unusual location of an epidermoid cyst occurring in the Sylvian fissure.
- There was a rare development of serious symptoms of an epidermoid cyst (signs of increased intracranial pressure, hemiparesis, and ataxia) that were completely resolved postoperatively.
- Imaging characteristics of an epidermoid cyst
- Details of the surgical resection procedure.
- Comparison with other studies of Sylvian fissure epidermoid cysts.

## Case report

**Figure 1.**
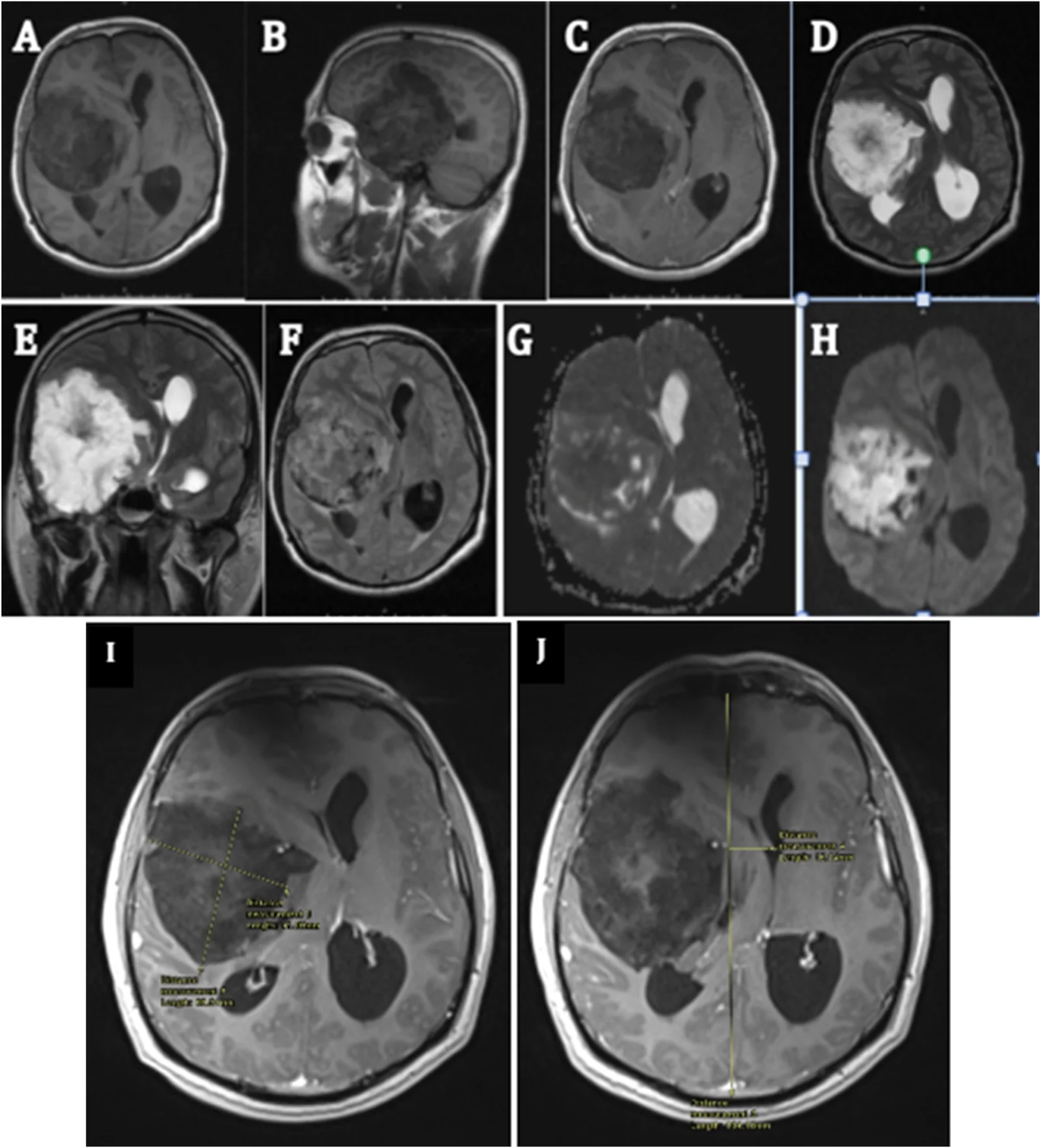
Axial (A) and sagittal (B) T1 images show a low-signal-intensity lesion with a few intrinsic high-signal foci, suggestive of a white epidermoid. Axial T1 post-gadolinium image shows no evidence of enhancement (C). Axial (D) and coronal (E) T2 images show a hyperintense lesion. Axial FLAIR image shows heterogeneous/dirty signal (F). Axial diffusion (G) and ADC (H) images show restricted diffusion. The size of the cyst (I) and the extent of midline shift (J) are depicted in axial sections.

A 41-year-old previously healthy man presented with a 1-year history of mild, paroxysmal headaches. He was evaluated at a polyclinic and diagnosed with a tension headache. Over several months, his headaches became more persistent, located in the right temporal region, associated with persistent nausea and vomiting, and did not respond to any medications. A few months after the clinical visit, the patient had a tonic-clonic seizure accompanied by urinary incontinence and a postictal phase that lasted for hours, leading him to the emergency department. On presentation to the emergency department, his vital signs and general physical exam were normal. The neurological exam demonstrated left-sided pronator drift, left-sided hemiparesis (4/5) in both upper and lower extremities, truncal ataxia, hyperreflexia of the left patellar, brachioradialis, and biceps tendons, and a positive Babinski sign on the left. Bilateral papilledema was present. Cranial nerve examination was unremarkable, and neither dysmetria nor dysdiadochokinesia was noted. Sensation was also intact. Brain magnetic resonance imaging (MRI) scans demonstrated a large, lobulated cystic lesion measuring 68.5 × 61.6 mm in the right Sylvian fissure extending into the frontal, insular, and temporal lobes with a midline shift of 16.14 mm to the left (Fig. 1). No hydrocephalus was noted. On magnetic resonance angiography (MRA), the cyst was in the right temporoparietal Sylvian fissure, encasing, without compromising, the branches of the right middle cerebral artery (MCA), and displacing the right MCA superiorly and medially, with the Sylvian vein lying superiorly. The cyst was hypointense on T1- and hyperintense on T2-weighted images, respectively, with areas of inhomogeneous hyperintensity on T1. It was also hyperintense on fluid-attenuated inversion recovery (FLAIR) imaging. When comparing diffusion-weighted (DW) sequences and apparent diffusion coefficient (ADC), the lesion was hyperintense on DW but hypointense on ADC, confirming restricted diffusion, a characteristic of an epidermoid cyst. The lesion did not show enhancement following intravenous contrast; these findings were compatible with a white cerebral epidermoid cyst, given the areas of inhomogeneous hyperintensity observed on T1 and restricted diffusion on DW/ADC (Fig. 1). The patient was advised to undergo surgical intervention, given the significant mass effect, neurological deficits, and symptoms, to which he readily agreed, and the surgery was performed during the same admission.

Neurosurgical approaches and microsurgical techniques

The neurosurgical approach to epidermoid cysts depends on tumor location and growth pattern. We will demonstrate the surgical approach for a Sylvian fissure epidermoid cyst. We used a standard microsurgical technique in this case, following these general steps.

### Positioning

Under endotracheal general anesthesia with the head flexed forward and secured in the Mayfield three-point fixation device rotated 30 degrees, the patient was positioned supine with the head turned to the left.

### Craniotomy and intraoperative findings

A craniotomy 6 × 7 cm craniotomy was performed. The operating microscope was introduced into the surgical field, and the operation proceeded with magnification ranging from 10 to 20×. The dura was opened by a horseshoe-shaped incision with its base toward the right anteriorly, and the dural edges were tented up. The tumor was then visualized under the arachnoid as a bright white lesion in the Sylvian fissure. The Sylvian fissure had already been opened by the cyst.

### Anatomical relations

**Figure 2.**
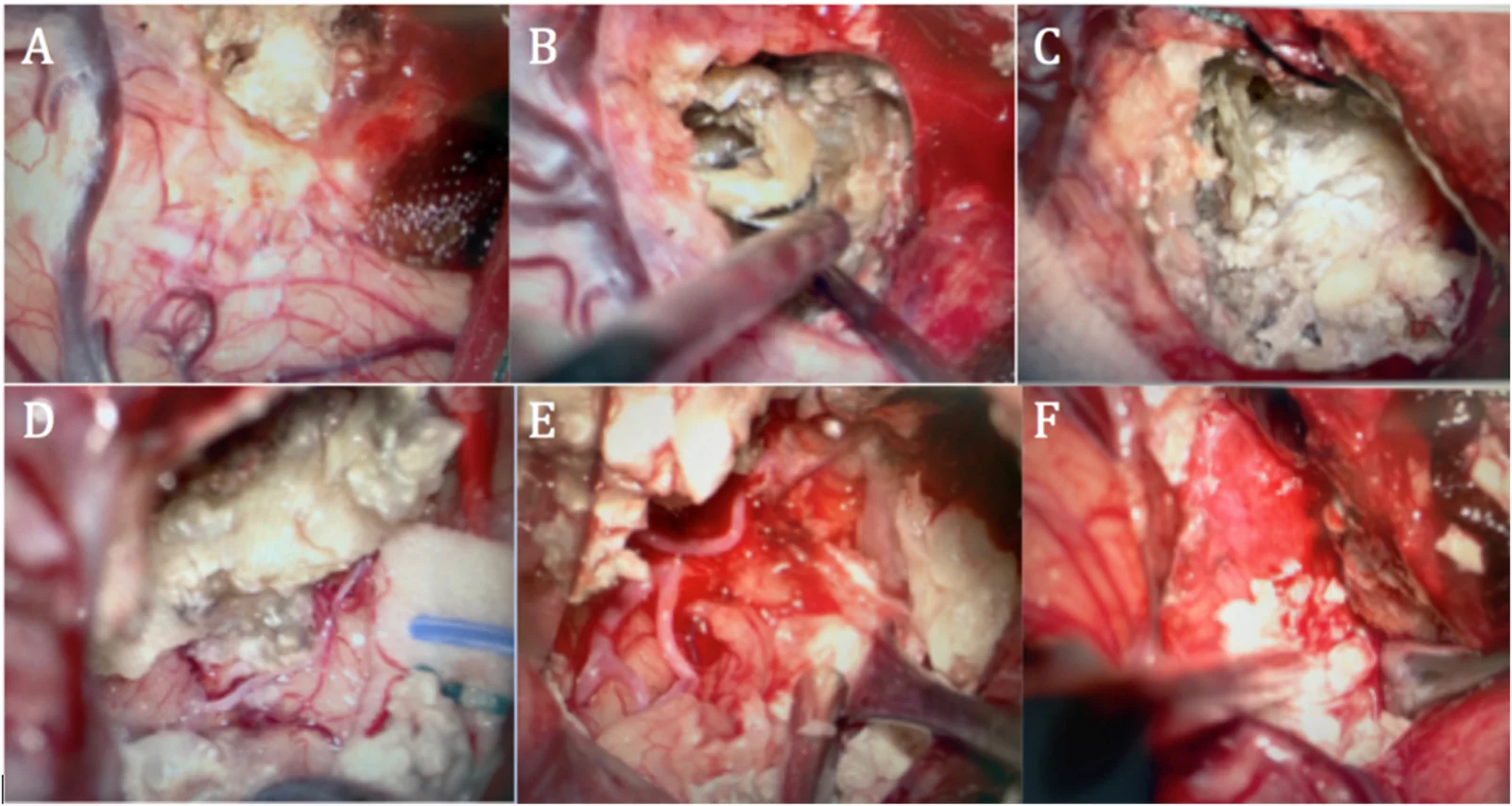
(A) Demonstrated the Sylvian vein pushed to the frontal lobe and not in the Sylvian fissure. (B) Opening the cyst and using the cupping technique for debulking the lesion. (C) Demonstrated the pearl appearance of the lesion. (D) Visualization of the temporal lobe. (E) Dissecting the MCA from the tumor capsule. (F) Demonstrated the visualization of the arachnoid space adhered to the capsule that was difficult to dissect and remove.

The Sylvian veins, lying superior to the cysts, were dislocated superiorly toward the right frontal lobe (Fig. 2A). The arachnoid attachments along the Sylvian fissure were opened, and the arachnoid membrane enveloping the tumor was incised from back to front along the Sylvian fissure using microscissors (Fig. 2B and C). After low-current bipolar coagulation of a few fine vessels traveling on the capsule, under saline irrigation, the lesion was incised, penetrated, and progressively debulked from within, mobilized, and removed in piecemeal cupping fashion. Careful attention was paid to identifying and preserving the arachnoid plane at the tumor-brain interface, which facilitated near-total resection and minimized small-vessel and brain injury (Fig. 2F). Medially, the cyst was related to the brainstem and the MCA.

### Tumor dissection

As the surgery proceeded within the space provided by progressive tumor debulking, the cyst was dissected and mobilized away from the MCA (Fig. 2E), insular cortex, and blood vessels by gentle, meticulous microsurgical techniques without applying any tension (Fig. 2B–E). At that point, we used fine micro scissors and several types of angled dissectors, in addition to multiple microscope angulations at varying magnifications. As tumor debulking proceeded by removing the epidermoid content, the brainstem progressively relaxed and provided additional working space for dissection. The lesion was mobilized and, once freed of adhesions, it was removed from the surgical field with cupped forceps without any complications; small fragments were aspirated with the aid of irrigation. We kept suction as low as possible during the microsurgical dissection to avoid injury to these fragile structures. A near-total resection was performed since dissection of the cyst capsule from the basilar and vertebral arteries, their perforating branches, the brainstem, or cranial nerves (CNs) could entail a risk of damage; therefore, we left a thin rim of tumor tissue attached to those structures.

During the operation, copious irrigation was performed, and cotton pads were placed around the exposed area to reduce spillage of irritating cyst contents into the subarachnoid space. Inspection of the tumor bed with the operating microscope is then performed to verify the extent of tumor resection.

### Post-operational images and follow-up

**Figure 3.**
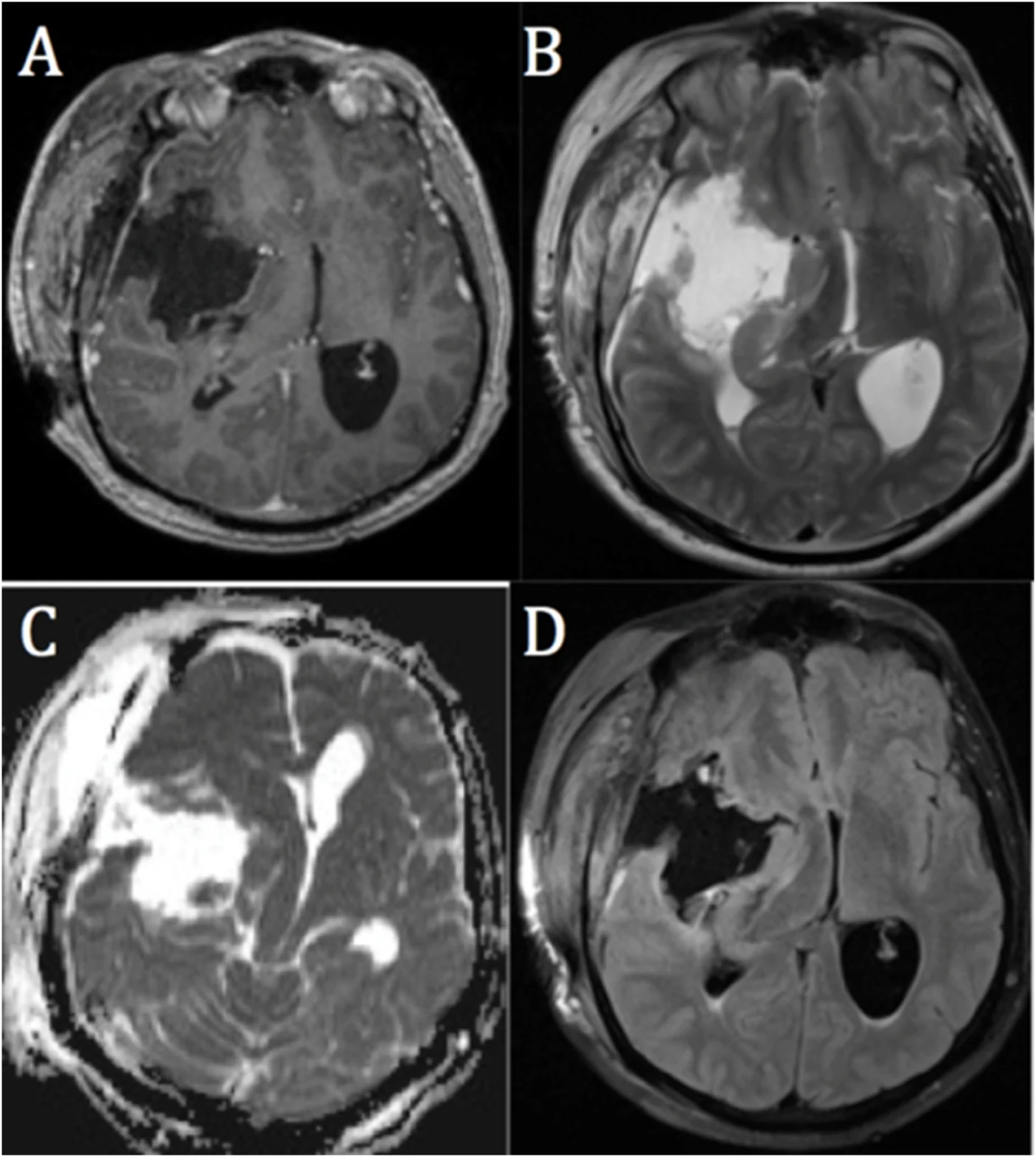
T1-weighted image (A), T2-weighted image (B), ADC (C), and FLAIR (D), showing near-complete resection postoperatively, with a small residual part shown on ADC (C) over the medial portion at the tumor location.

**Figure 4.**
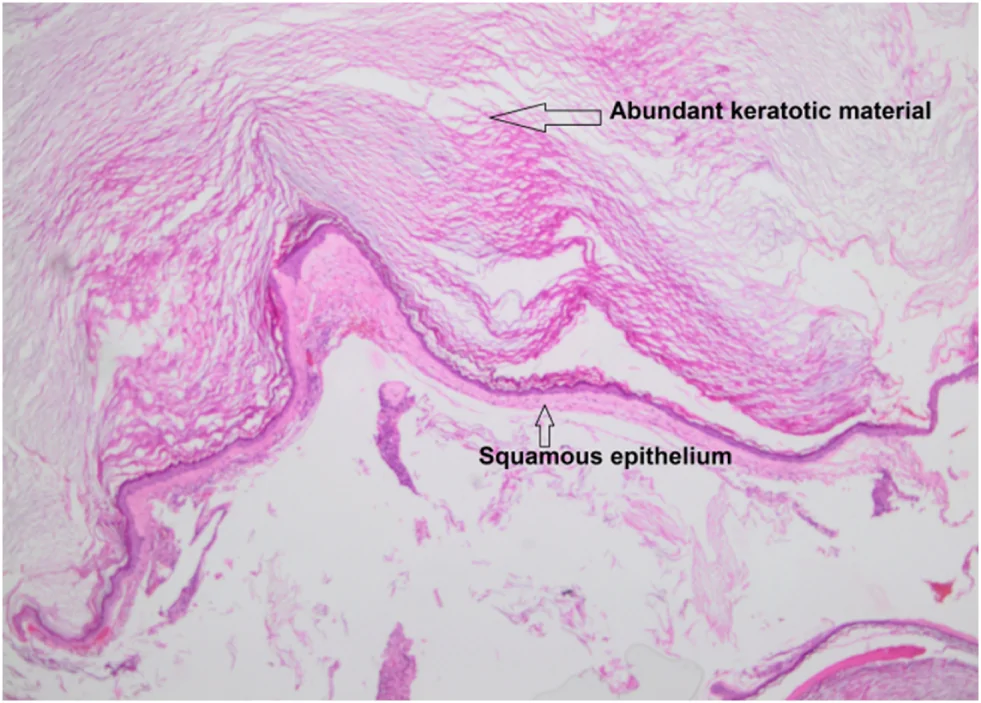
Histopathological microphotograph demonstrating a stratified epithelium wall (black arrows) containing keratin and cholesterol characteristics of an epidermoid cyst, (hematoxylin and eosin stain, ×100 magnification).

Post-op images, along with pathology images, are provided in Figures 3 and 4 respectively. The patient had complete resolution of symptoms after resection during follow-up visits with the attending physician, which were performed 2 weeks post-op, at 6 months, and then repeated yearly. Five years later, the patient still had a normal neurological examination. The last MRI, performed on 3/7/2025, showed stable findings without interval change.

## Discussion

Epidermoid cysts are believed to originate from remnants of embryonic ectodermal tissue around 3–5 weeks of gestation^[^6,7^]^. Epidermoid cysts are typically considered congenital, forming during the third to fifth weeks of intrauterine life due to abnormal trapping of ectodermal cells. Over time, these cells give rise to epidermis within nervous tissue during neural tube closure^[^8^]^. During this embryonic period, the otic and optic vesicles also take shape. The presence of ectodermal cells within these structures is associated with epidermoid cysts in the cerebellopontine angle and parasellar region, which are common sites in the brain^[^9–11^]^. Other locations where these cysts may occur include the parapontine region, middle cranial fossa, parapituitary region, diploë, and spinal canal, although purely intracerebral epidermoids remain rare^[^11–13^]^. Epidermoid cysts typically remain asymptomatic due to their gradual growth. However, after several decades of life, they may lead to insidious mass effects, with or without cranial nerve involvement. This mass effect arises from the accumulation of cyst contents, which can directly compress brain structures^[^8,14^]^. Their enlargement results from the buildup of keratin and cholesterol, originating from the shedding of epithelial cells. However, their delayed presentation may be linked to the lack of significant mass effect, as these tumors tend to grow within available cisternal spaces^[^15,16^]^.

The most common locations of intracranial epidermoid cysts are as follows: the cerebellopontine angle (CPA), cerebellopontine cisterns, parasellar region, tentorium, middle cranial fossa (MCF), and, finally, the ventricles^[^6,17–19^]^. Epidermoid cysts are usually well-circumscribed, with a complete capsule, and are slow-growing cysts with an insufficient blood supply^[^6^]^. On imaging, epidermoid cysts appear as hypointense lesions on T1-weighted MRI and hyperintense on T2-weighted imaging, and they show true diffusion restriction^[^20,21^]^.

Only a few cases of Sylvian fissure epidermoid cysts have been reported. Compared with our case, which presented with signs of increased intracranial pressure (headache, nausea, vomiting, bilateral papilledema) along with truncal ataxia and a seizure associated with mild left hemiparesis, other studies reported progressive aphasia and intention tremor, which were not observed in our patient^[^20,22^]^. Moreover, only our case showed an abnormal neurological finding on physical examination (a pronator drift), whereas other case reports showed no neurological abnormalities. However, headache has been a common finding^[^22,23^]^. Table 1 summarizes the case reports on Sylvian fissure epidermoid cysts.

**Table 1:** summarizing other reports of Sylvian fissure epidermoid cysts.

Article/ year	Age	Sex	Symptoms	Image	Extent of resection	Post-op	Follow-up
G. Zenonos *et. al*^[^8^]^/2018	53	M	-Gradual slowing of cognitive speed	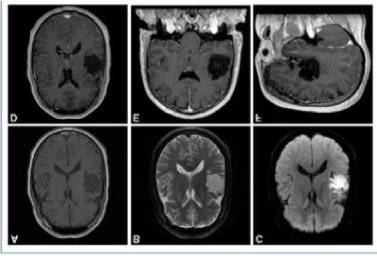	Near-total excision with only small capsule remnant	Symptom-free	6 M
			Progressive headaches Progressive aphasia Transient blurred vision				
Shah *et al*^[^10^]^	24	M	-Increasing intentional tremor of left limbs	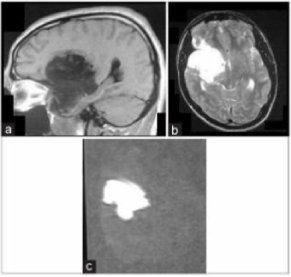	Near total excision	Tremor decreased then disappeared over 3 M	1 year
			Mild headache				
Gosal *et al*^[^11^]^/ 2019	30	F	Headache	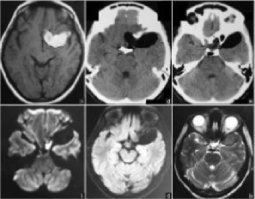	Gross total excision	Symptom-free	6 M

## Conclusion

Our case presents a rare location of an intracranial epidermoid cyst causing neurological deficits impacting the patient’s quality of life, which completely resolved post-resection. We have provided accurate imaging and histological results confirming the diagnosis, along with a step-by-step guided surgical procedure during which we left a small part of the capsule on eloquent structures to avoid the risk of harming the patient.

## Data Availability

Provided upon request as per confidentiality agreement.

## References

[1] Kato K Ujiie H Higa T. Clinical presentation of intracranial epidermoids: a surgical series of 20 initial and four recurred cases. Asian J Neurosurg 2010;5:32–40. PMC3198667 22028741

[2] Toglia JU Netsky MG Alexander E. Epithelial (Epidermoid) tumors of the cranium. J Neurosurg 1965;23:384–93. 10.3171/jns.1965.23.4.0384 5853888

[3] Alvord EC. Growth rates of epidermoid tumors. Ann Neurol 1977;2:367–70. 10.1002/ana.410020504 617575

[4] Menon B Sasikala P Agrawal A. Giant middle fossa epidermoid presenting as Holmes’ tremor syndrome. J Mov Disord 2014;7:22–24. 10.14802/jmd.14005 PMC4051724 24926407

[5] Kerwan A Al-Jabir A Mathew G. Revised Surgical CAse REport (SCARE) guideline: an update for the age of artificial intelligence. Prem J Sci 2025;10:100079.

[6] Cheng Z Fan SZ Sun YX. Lateral intraventricular epidermoid cyst: a case report and literature review. Neurol India 2023;71:1002–06. 10.4103/0028-3886.388112 37929444

[7] Zhou F Yang Z Zhu W. Epidermoid cysts of the cavernous sinus: clinical features, surgical outcomes, and literature review. J Neurosurg 2018;129:973–83. 10.3171/2017.6.JNS163254 29271707

[8] Patibandla M Yerramneni V Mudumba V. Brainstem epidermoid cyst: an update. Asian J Neurosurg 2016;11:194–200. 10.4103/1793-5482.145163 PMC4849286 27366244

[9] Gopalakrishnan CV Dhakoji A Nair S. Epidermoid cyst of the brainstem in children. J Child Neurol 2012;27:105–12. 10.1177/0883073811414709 21862831

[10] Czernicki T Kunert P Nowak A. Epidermoid cysts of the cerebellopontine angle: clinical features and treatment outcomes. Neurol Neurochir Pol 2016;50:75–82. 10.1016/j.pjnns.2015.11.008 26969562

[11] Hu X-Y Hu C-H Fang X-M. Intraparenchymal epidermoid cysts in the brain: diagnostic value of MR diffusion-weighted imaging. Clin Radiol 2008;63:813–18. 10.1016/j.crad.2008.01.008 18555040

[12] Labuschagne J Mutyaba D Koranteng P. Intrinsic brainstem epidermoid: case report and literature review. Wits J Clin Med 2020;2:199–202.

[13] Kaido T Okazaki A Kurokawa S-I. Pathogenesis of intraparenchymal epidermoid cyst in the brain. Surg Neurol 2003;59:211–16. 10.1016/s0090-3019(02)01042-x 12681557

[14] Hasegawa M Nouri M Nagahisa S. Cerebellopontine angle epidermoid cysts: clinical presentations and surgical outcome. Neurosurg Rev 2016;39:259–67. 10.1007/s10143-015-0684-5 26566990

[15] Agha RA Borrelli MR Farwana R. The PROCESS 2018 statement: updating consensus preferred reporting of case series in surgery (PROCESS) guidelines. Int J Surg 2018;60:279–82. 10.1016/j.ijsu.2018.10.031 30359781

[16] Chowdhury F Haque M Sarker M. Intracranial epidermoid tumor; microneurosurgical management: an experience of 23 cases. Asian J Neurosurg 2013;8:21–28. 10.4103/1793-5482.110276 PMC3667457 23741259

[17] Pamir MN Kilic T Özek MM. Non-meningeal tumours of the cavernous sinus: a surgical analysis. J Clin Neurosci 2006;13:626–35. 10.1016/j.jocn.2006.04.004 16860718

[18] Dufour H Fuentes S Metellus P. [Intracavernous epidermoid cyst. Case report and review of the literature]. Neurochirurgie 2001;47:55–59. 11283457

[19] Li F Zhu S Liu Y. Hyperdense intracranial epidermoid cysts: a study of 15 cases. Acta Neurochir (Wien) 2007;149:31–39. 10.1007/s00701-006-1060-6 17151831

[20] Zenonos GA Cabral DTF Olexa J. Left sylvian fissure epidermoid cyst presenting with progressive aphasia. World Neurosurg 2018;120:363–67. 10.1016/j.wneu.2018.08.164 30172982

[21] Karantanas AH. MR imaging of intracranial epidermoid tumors: specific diagnosis with Turbo-FLAIR pulse sequence. Comput Med Imaging Graph 2001;25:249–55. 10.1016/s0895-6111(00)00069-0 11179701

[22] Shah A Makkiyah F Goel A. Sylvian fissure epidermoid cyst presenting with intention tremor. Asian J Neurosurg 2016;11:174–75. 10.4103/1793-5482.175620 PMC4802947 27057232

[23] Gosal J Joseph J Khatri D. White epidermoid of the sylvian fissure masquerading as a dermoid cyst: an extremely rare occurrence. Asian J Neurosurg 2019;14:553–56. 10.4103/ajns.AJNS_241_18 PMC6515996 31143281

